# Toleration and Pragmatism: Themes from The Work of John Horton

**DOI:** 10.1007/s11406-016-9767-y

**Published:** 2016-11-05

**Authors:** Sorin Baiasu

**Affiliations:** Keele University, Keele, UK

**Keywords:** Toleration, Modus vivendi, Pragmatism, Realism, Contigency, Political, Obligation

## Abstract

John Horton’s work has been particularly influential in debates on specific topics related to toleration, political obligation, modus vivendi and political realism. More recently, he has synthesised these views in the form of a distinctive position in political philosophy, a position that has the potential to question much of the received wisdom in the field. The papers of this special issue engage with some of the most fundamental issues of Horton’s account, more exactly, the related issues of toleration and modus vivendi, Horton’s account of associative obligations, with a focus on the methodological assumptions which underpin his position more generally, and the metaphysical presuppositions of his account, in particular, the presupposition of contingency. I offer brief presentations of the papers in the special issue and of the ways they link with each other. In the discussion of the papers by Forst, Newey, Jones, Weale and Mendus, the emphasis will be on those arguments which question Horton’s position. Horton’s paper will then be presented with a focus on possible responses to these challenges. I will conclude with several remarks on an unexpected continuity between Horton’s realist view and a view realists usually criticise as idealising, namely, John Rawls’s theory of justice as fairness.

## Introduction

A tolerant attitude combined with a pragmatic approach would seem to be the ideal strategy to be pursued in politics today. They would oppose the increasingly sectarian reactions of politicians and members of the public, and would temper the fanatism with which certain principles and values are often pursued. Toleration allows a person to accept other practices than the ones with which she is accustomed, although these are practices to which she objects. Pragmatism enables parties in conflict to co-exist even when the accepted framework for their co-existence is not regarded as entirely just, but perhaps as a second-best, a modus vivendi. Underlying these qualities, there seems to be a realist perspective on politics, a perspective which puts emphasis on the observation of the particular, the preservation of relevant differences and the significance of the contingent circumstances of the concrete situation in which we find ourselves.

In his work, John Horton has presented, discussed and defended these ideas, some of their philosophical presuppositions and their ethical, political and social implications. His work has been particularly influential in debates on specific topics related to toleration, political obligation, modus vivendi and political realism.^1^ More recently, however, he has also synthesised these views in the form of a distinctive position in political philosophy, a position that has the potential to question much of the received wisdom in standard political theory. The papers of this special issue engage with some of the most fundamental issues of Horton’s account, and question its cogency and implications.

Briefly presented, the foci of the papers are as follows: the first three papers discuss the related issues of toleration and modus vivendi, either by focusing on one of them individually (Forst, on toleration, whereas Jones, on modus vivendi) or on their relation (Newey). The next paper of the special issue (Weale’s) discusses Horton’s account of associative obligations, but with a focus on the methodological assumptions which underpin Horton’s position more generally. Finally, Mendus’s piece continues the discussion on methodology, but with an emphasis on an even more fundamental aspect of Horton’s theory, namely, the metaphysical presuppositions of his account, in particular, the presupposition of contingency. Horton’s article concludes this special issue and can be read both as a response to some of the issues raised in the papers by Forst, Newey, Jones, Weale and Mendus, on the one hand, and, on the other, as a further elaboration on his own position.

In what follows, I will offer brief presentations of the papers and of the ways they link with each other. In the discussion of the papers by Forst, Newey, Jones, Weale and Mendus, the emphasis will be on those arguments which aim to question Horton’s position. Horton’s paper will then be presented with a focus on possible responses to these challenges. I will conclude with several remarks on an unexpected continuity between Horton’s realist view and a view realists usually criticise as idealising, namely, John Rawls’s theory of justice as fairness.

## Toleration

Forst aims to offer an immanent critique of Horton’s account of the virtue of toleration. He begins with a presentation of the conceptual framework, continues with a discussion of the differences between his and Horton’s approaches and concludes with an argument against a tension in Horton’s account.

As any student of toleration finds out quite early on, toleration has three dimensions. First, a practice or belief has to be judged as false or bad in order to be a candidate for toleration. This is the objection dimension. Secondly, if this practice or belief which is objected to is to be tolerated, there must be some reasons why it would be wrong not to tolerate it. This is the acceptance dimension. The reasons for the acceptance of a belief (or practice) do not eliminate the reasons for objecting to the belief, but trump them. Finally, there are reasons for rejection which mark the limits of toleration; in other words, certain beliefs or practices (say, racist) must be rejected, rather than being simply objected to and accepted. This is the dimension of rejection.

On Forst’s account, toleration is a normatively dependent concept – it needs other normative resources in order to acquire content. He thinks he differs from Horton in the way in which they understand how toleration is provided with content along the three dimensions. The common background for Forst and Horton is a respect conception of toleration. According to the respect conception, distinct groups in society should recognise each other as consisting of equal citizens; all groups (both the majority and minorities) should have equal legal and political status. Groups may differ considerably in their cultural practices and they may hold conceptions of the good life which are incompatible, but they accept a common framework consisting of norms which do not unfairly favour a particular group.

Another common element of Forst’s and Horton’s accounts becomes evident in a discussion of the paradoxes of toleration. Thus, the first paradox is that a racist who would not act on his beliefs for certain reasons, including strategic ones, would be seen as tolerant. Yet, if toleration is a moral virtue, then the objection to the belief or practice to be tolerated cannot be based on prejudices or hatred. Following Bayle, Forst is in agreement here with Horton, who supports a strongly rationalist argument. For Horton, the objection to the practice to be tolerated must not be unreasonable or without value.

What seems to differentiate Horton’s view from Forst’s Bayle-inspired conception becomes visible when a second paradox of toleration is discussed. Thus, according to this paradox, we are supposed to object to and accept the same practice. Horton accounts for this by reference to autonomy. It is an expression of autonomy for a person to perform a particular practice or hold a certain belief, even when this is the wrong one. Yet, Forst argues, if a practice is morally wrong, the fact it was chosen freely may suggest it is appropriate to reject, rather than tolerate, it. Secondly, a liberal (of the perfectionist stripe, for instance) may value autonomy only when it issues in good choices. Furthermore, practices or beliefs which result from traditions or faith would not be good candidates for toleration, since their toleration would be justified by reference to autonomy, while an implication of their toleration is that autonomy is undermined. Finally, an argument for toleration based on a particular conception of the good (the liberal value of autonomy, for instance) would be insufficient – a non-partial argument would be needed.

By contrast, Bayle’s argument for toleration tries to provide precisely such a non-partial justification. At this juncture, the tension in Horton’s argument starts to become visible. While he seems to answer the first paradox by reference to such a non-partial justification (which would rely on reasons which are not unreasonable or without value), he thinks that there is no non-partial justification of toleration acceptable to all. The same tension, Forst argues, is generated by Horton’s response to the third paradox, according to which unlimited tolerance leads to the disappearance of toleration. Horton’s answer is that toleration cannot require that anything be tolerated, and there are necessary limits (provided by moral standards) beyond which a practice or belief must simply be rejected.

An illuminating excursus in the history of philosophy, with particular reference to Bayle’s answer to Augustine’s peculiar argument for intolerance, is the next step in Forst’s argument. Particularly important here is Bayle’s view of the independence of morality from faith. Whereas reason conveys the principles of logic, metaphysics and morality, religious conscience is crucial for issues of faith and salvation. He thinks universal moral precepts should not be violated by any interpretation of religious texts. He argues against the view that what is unjust can become just by being done in favour of a true religion. This is not because he would be sceptical of religious truths, but because he thinks our rational capacities are limited: our epistemic capacities help us to come to a firm view of religious matters, but not to establish this view as the only true one on the basis of objective reasons.

Forst focuses next on a discussion of Bayle and Horton. He takes Bayle to use reflexively the principle of justification as the ground for the justification of toleration. The question of justification is the question of the legitimacy of the use of force and of general standards; it can be seen as a moral principle of mutual respect, which presupposes a standard of reciprocal justification. As a result, a religious practice or belief can be objected to by those who do not belong to the particular religious group. Given that it will form the object of reasonable disagreement, however, it can be accepted on the basis of a duty of self-restraint in the face of reasonable disagreement. In other words, when it is reasonable to disagree with regard to a particular practice, there is a duty of self-restraint which protects that practice, in spite of the disagreement. Finally, all practices or beliefs which violate the principle of equal respect are not simply objected to, but rejected and they constitute the limits of toleration.

Hence, Forst notes, like Horton, Bayle distinguishes between reasonable and unreasonable objections to practices or beliefs, but, unlike Horton, he thinks theoretical and practical principles have a validity which goes beyond reasonable disagreement. Horton sees disagreement as going all the way down and affecting all normative claims; he does not simply assert value pluralism as a fact of our day-to-day existence, but as an implication of the limits of reason, which is unable to provide content to our normative requirements. Forst concludes with the formulation of another paradox of toleration, “Horton’s paradox”: asking persons to reasonably check whether their objections are based on prejudice, but without asking them to respect others by providing mutually acceptable reasons for common norms. It is paradoxical to argue for toleration as a moral value or virtue and at the same time to leave open what counts as an appropriate moral reason for that value or virtue. Without this element of normative validity, Horton would be forced to leave the respect model of toleration, which he seems to share, and would be forced to be content with a modus vivendi. For the purpose of Forst’s argument, this conclusion is sufficient, but the paper does also raise the question of the relation between toleration and modus vivendi. This will be the topic of the next section.

## Toleration and Modus Vivendi

At the beginning of his article, Newey identifies three parts of Horton’s work: the first one, on toleration, the middle one, on associationist political obligation and the third one, on modus vivendi. Yet, Newey writes, one feature of toleration which seems to meet an almost universal agreement is the condition (called also the ‘Power Condition’^2^) that the tolerator have the power not to tolerate the tolerated’s objected-to practice or belief. A racist would gladly repatriate all immigrants had she the power to do this, and the fact that she does not perform this racist act, because she does not have the power to do so, shows that her acceptance of the immigrants is not the result of a tolerant attitude.

But, if on a standard reading a modus vivendi is the result of two or more parties agreeing on a compromise, because none has the necessary power to impose its preferred arrangement, then the compromise is not a sign of toleration, but of powerlessness. This seems to suggest that toleration and modus vivendi are compatible only on a non-standard understanding of at least one of them. The focus of Newey’s piece is on toleration and he eventually aims to improve on the standard account of toleration, rather than propose a non-standard one. As we will see later on, this leaves open the possibility for Horton to articulate a non-standard conception of modus vivendi, if this defence of the virtue of toleration is to be compatible with a modus vivendi.

Newey shares with Horton a general suspicion of theory in the study of politics. More exactly, they are aware that theory can easily start from assumptions or adopt strategies, which do not correspond to facts not only for some contingent reason, but for necessary considerations related to their idealizing character. One explicit aim of Newey’s essay is to explore one of the methodological strategies of idealizing theories (namely counterfactual reasoning) and to indicate its limits in discussions of toleration.

In the way in which PC was formulated above, it does not take into consideration a situation where an agent wrongly believes she has the power to prevent the disapproved-of practice or belief, but she still acts tolerantly. Furthermore, as formulated above, PC does not account for a situation where, although a person could prevent the objected-to action, she does not prevent it because of the high costs involved in the prevention of the practice. Both of these are not cases of toleration, although they are cases of self-restraint where the agent has the power not to accept the objected-to practice or belief. Assuming that PC is reformulated to account for these cases, there is still the issue of the distinction between considerations of costs and principles. We can, for instance, talk about the moral costs of breaking a principle, in which case, we can have a situation of toleration where the objected-to practice or belief was not prevented, because preventing it would have meant incurring undue moral costs. This seems to exclude it as a case of toleration, if we take into consideration the second problem I have just mentioned (namely, that not preventing a objected-to action because of the high costs of prevention does not count as a case of toleration).

Let us assume that we find an appropriate way of distinguishing between acting on a principle and acting in order to avoid certain costs, and that a formulation for PC can subsequently be offered.^3^ The significance of toleration, Newey claims, lies in the underlying attitude of the agent. This attitude can be examined by using counterfactuals. The test for the identification of the attitude of an agent starts from the assumptions that the agent believes he has the power to prevent the objected-to action and he does not believe prevention incurs high costs, and the question is whether he would try to prevent it; if he did, we would not regard his non-prevention as tolerant; if he did not, then we could say he would be committed to an acceptance principle which supports toleration.

However, using the counterfactual strategy, it may seem possible to idealise principles away. In fact, according to Newey, principles are immune to counterfactual critique and it is in this way that we can distinguish them from costs. Principles themselves go to determine what the counterfactual ideal is. For instance, during the World War II, the Nazis could have diverted further resources from other fronts to track down and kill yet more Jews. The fact that the Nazis failed to devote more resources to genocide does not indicate tolerance of the remaining Jews; it was not on the basis of a principle, such as the absolute value and equality of human lives, that the Nazis decided not to devote further funding, but due to rising marginal costs.

One problem Newey raises about the application of counterfactual reasoning concerns the possibility of imagining that I have an extraordinary power which enables me to bring about a world in which objected-to practices or beliefs do not occur. In such a world, there would be no need for toleration, since there would be no practice or belief of which I would disapprove. Yet, this shows one limit of the counterfactual critique: the demand that real-world commitments be checked against counterfactual scenarios represents an error realist critics of liberalism have already targeted. The world does contain bad things. According to theodicy, the bad things are a necessary consequence of our free will. This, for Newey, seems to suggest a basis for non-interference, namely, the value of freedom. The freedom on the basis of which the tolerator extends toleration must be such that he is prepared to accept that the other person will act in ways of which he disapproves and he will let her do so anyway.

Yet, this idea of freedom is considered by Newey too thin to gain political traction. This is a politically relevant idea, but needs a normatively more substantial account of circumstantial freedom; in this way, specific decisions can be evaluated as tolerant or intolerant. Moreover, according to Newey, once the notion of toleration is no longer considered as positive, it can be used to refer to self-restraint in conditions of mutual attrition, as is the case of a modus vivendi. Yet, such a ‘neutral’ notion of toleration would be based on a radical notion of freedom, one which would allow the tolerator to renounce even principles by which counterfactuals ideals are framed. With such a notion of freedom, however, one could argue that there is no toleration.

The analysis of toleration provided so far, Newey concludes, shows that power is an ineradicable feature of the political life, while at the same time acknowledging the limits on what we have the power to do. The problem is, Newey claims, that agents are not free in a radical sense. Agents are not free to renounce their ethical principles or their ethical dispositions. This means that the tension between modus vivendi and toleration will persist, unless a distinct, non-standard notion of modus vivendi will be adopted. In the next section, we will look more closely at the notion of a modus vivendi.

## Modus Vivendi

In his contribution to this special issue, Jones starts by presenting Horton’s political theory of modus vivendi as placed between two extreme positions: a regime of tyranny and suppression, on the one hand, and, on the other, a regime of justice, where citizens perceive their political organization as just and do not experience conflicts between their comprehensive doctrines and their society’s public institutions. Unlike a regime of tyranny and oppression, a modus vivendi values the citizens’ consent, acceptance or agreement. Unlike a regime of justice, a modus vivendi does not set very strict conditions on which forms of consent, acceptance and agreement are to be considered genuine. Moreover, compared to the regime of justice, a modus vivendi includes an element of compromise and the feeling that it is only a second-best arrangement.

This characterisation of modus vivendi allows for variations in the way in which it is presented by political theorists. For instance, according to Rawls (as a supporter of a regime of justice), a modus vivendi rests entirely upon a mere balance of powers, which is formed between the self-interests of the members. By contrast, for Horton, a modus vivendi need not be seen as constructed entirely on the basis of self-interest, but can be conceived of as built on anything the parties can contribute. For instance, moral beliefs may make easier the construction of, and may consolidate, a modus vivendi. Unlike a regime of justice, moral beliefs are in this case considered instrumentally, that is, insofar as they can lead to a more stable modus vivendi, and the modus vivendi itself is not justified on the basis of these moral beliefs. Moreover, Horton’s modus vivendi is not considered legitimate due to its intrinsic normative features, but because of the way it is regarded by its population. The legitimacy of this type of modus vivendi is less demanding than that of a just regime, and this seems to be right: a population may accept a political arrangement as legitimate even if they think it is not fully just. Thus, Rawls tries to adjudicate conflicts between comprehensive conceptions of the good in a just way and in a way recognizable by citizens as just; by contrast, Horton’s modus vivendi has an interest in justice, but the aim is that the political arrangement be not significantly unjust.

One important aspect of Horton’s modus vivendi is its normative status, and Jones focuses on this next. One possibility is that Horton’s modus vivendi is considered a better description of the political reality of liberal democracies. Another possibility is that it is regarded as a model to be followed by liberal democracies. The latter interpretation is supported by the fact that Horton’s modus vivendi is presented as superior to a tyrannical regime; yet, this is not much support, since a tyrannical regime may be objectionable on simply conceptual considerations. Moreover, in some situations, a tyrannical regime may deliver more easily peace and security. The notion of legitimacy may mean that a particular regime is accepted by the population or that it is deserving of compliance. Horton seems to have in mind the latter, although he also suggests the notion is inherently normative. Jones concludes that this issue is left open in Horton’s account of a modus vivendi. His notion of legitimacy may be permeated with normativity, but it is not directly prescriptive in purpose and does not aim to provide much practical guidance.

On Jones’s view, Horton’s advocacy of the modus vivendi may seem prompted by several considerations. First, there is some modesty about what we can expect from human beings, given their limited abilities and cognitive capacities. Secondly, in politics, we start from a particular context or particular circumstances, something which precludes the realization of neat models of a just society. Thirdly, morality is a matter of dispute, rather than a way to solve disputes – and this applies even to higher-order principles of justice. Legitimacy, as part of Horton’s modus vivendi, depends on culture and context, as well as on what the relevant population finds acceptable.

Concerning the relation between modus vivendi and actual political systems, Jones notes that a modus vivendi is not intended only for the special circumstances of deeply divided societies. Nevertheless, reasonably stable democracies seem to be based on a consensus on basic principles of the political system which goes deeper and is wider than in a modus vivendi. If we place a modus vivendi at the level of specific issues, then disagreement is more visible, but then the modus vivendi arrangements become an omnipresent feature of politics, and modus vivendi loses any significant independent meaning. Jones concludes that the test should not be the level of disagreement, since in time citizens may no longer feel any disagreement with regard to how their political systems solve conflicting claims, although the system is a modus vivendi.

Finally, Jones examines how far Horton’s modus vivendi is from contemporary political theory. On Jones’s account, Rawls’s later theory of justice can be considered as ‘realist’, in particular his view that principles of justice are to be supported by ideas that are already present in the public culture of democratic societies seems attentive to context and detail. Rawls’s distinction between ideal and non-ideal theory is also clarified by Jones as having a distinct function, and one which by no means entails that Rawls’s account would have an ideal character. Moreover, Rawls describes his account of a just international order as a realistic utopia, although many commentators have found it more realistic than utopian. Rawls’s later theory seems no more ideal than other normative accounts, which also aim to be prescriptive.

The following critical comments formulated from the perspective of the modus vivendi seem also to suggest a significant difference between Rawls’s theory and Horton’s modus vivendi. Thus, first, the Rawlsian model of the overlapping consensus may seem appropriate for the relation between religion and power, although not yet for that between power and other comprehensive doctrines. Secondly, Rawls suggests sometimes that citizens not only embrace his theory, but also endorse it within their different comprehensive doctrines, which is unnecessarily demanding. Thirdly, the stability that preoccupies Rawls is intellectual as much as political, and, again, the concern should be primarily political.

Yet, Jones notes, these objections do not provide indirect support for a modus vivendi, but rather show that the political arrangements proposed by Rawls seem to be more easily realizable than Rawls suggests in theory. Rawls’s focus on the differences of doctrines between the citizens may be an appropriate point to object to, although the focus could be changed from this type of difference to a difference of identities. Horton also thinks Rawls ignores questions of political judgement, leadership, representation, political responsibility, what is politically possible and the transition from existing societies to just societies. Jones replies, however, that part of Horton’s criticism (for instance, that concerning representation) is misplaced, part (on leadership), justified by modesty of ambition, whereas Horton’s dismissal of the significance of normative preoccupations deprives political theory of anything significant or distinctive to say. What remains problematic, Jones concludes, is Horton’s skepticism about the normative role of political theory and his view that politics is so sui generis that political theory is unable to provide practical guidance, and this seems to be also a fundamental difference between Rawls’s theory and Horton’s modus vivendi. Additional problems seem to be raised by Horton’s methodology, which will be the focus of the next section.

## Methodology

Weale’s paper begins with an examination of Horton’s account of political obligation. This consists of the conjunction of two claims. First, there is the claim that political obligations are concomitant of membership of a particular polity. Secondly, there is the claim that a polity is a form of association that has as generic value the goods of order and security. These claims are justified empirically as follows. First, as a matter of fact, most people living in stable polities will think of themselves as having a special connection with their polity. This makes political obligation a concomitant of membership of a particular polity. Secondly, polities are forms of association with a unique ability to supply the goods of order and security. If this is correct, then obligation is neither voluntary nor owed to the world at large, but it is incurred as an incident of membership and it owed to a particular polity.

Weale’s claim is that Horton’s account of political obligation needs to be supplemented by an account of the reasons justifying to individuals why they should play their part in any scheme of cooperation necessary to produce the social goods of law and order. This is the claim which introduces the methodological concerns of the paper. Thus, according to Weale, once it becomes evident that the account of the reasons justifying participation is a necessary part of an account of political obligation, Horton’s methodology appears as no longer sufficient.

To clarify how he understands Horton’s methodology, Weale places it between two paradigmatic extremes: at one end, the demonstrative method, which starts from a limited number of premises and axioms from which conclusions are drawn through a deductive process of reasoning (as in David Gauthier); at the other end, the oracular method, used by theorists of discontent, who are hostile to a deductive style of reasoning and prefer an aphoristic, poetic and provocative manner (Nietzsche seems to be a good example here). Horton’s methodology, Weale explains, shares with the oracular methodology a skepticism about the reach and scope of deductive reasoning; yet, unlike the oracular methodology, it does not try to provoke, but to seduce.

Both extreme strategies dispense with any attempt to ground normative arguments in an understanding of ordinary moral consciousness, denying a place for reflective equilibrium in their theorizing. While, on Weale’s account, Horton regards the content of ordinary moral consciousness as offering a constraint on what we can say in our theories, he nevertheless denies a place for reflective equilibrium, since this method presupposes the distinction between abstract theory and the contents of ordinary moral consciousness, whereas Horton rejects the appeal of abstract theory.

Given the claims of Horton’s account of political obligation and his empirical justification of these claims, it seems his methodology is able to explain the standard case of political obligation (a), namely, the case where people are born into a polity; moreover, it can deal with cases where obligation is significant (b) and meets the requirement of showing how political obligation is owed to a particular polity (c). (a), (b) and (c) are desiderata for a theory of political obligation, and the methodology of Horton’s account seems able to meet them. One puzzling feature of Horton’s methodology, Weale notes, is his claim that he uses interpretation to provide justification in the manner of a philosophical explanation. Given the way interpretation and explanation are usually contrasted in the literature on methodology, Weale suggests the following construal of Horton’s claim: he regards Horton’s reference to interpretation as an indication that he is using a sort of logical analysis, which explains the meaning of concepts and draws their implications, leading in this way to understanding.

Thus, Horton’s phenomenology would focus on the concepts present in the mental life of the members of the society and would show that these members feel a special association with their polity, while at the same time accommodating a critical attitude. The advantage of the appeal to the facts of common moral consciousness is that, unlike other strategies based on abstract concepts (for instance, tacit consent or hypothetical contract), these are facts which do not need deep thinking and will not lead to implausible results.

The problem is the following: by reflecting on the fact of the attachment of the members of a society to that society’s institutions and practices, members of the society should get to believe that they have reasons for accepting their political obligations, given the community’s capacity to provide security and order. And, yet, the question is precisely how this will happen. The problem of political obligation, as presented by Weale, is whether there really is a reason why I should act in ways in which so far I have thought I ought to act, a question formulated by Prichard. There are various ways to deal with this question, for instance, to reject the question or to assume it has already been answered. Yet, granting with Weale that it is a genuine question, the further issue is that some members of a society may not find their membership as a sufficient reason for their political obligations. Although they accept that their community provides the goods of order and security, they may still prefer to free ride.

The kind of reason the dissenter would need would be a justification which would show that cooperative action can be individually beneficial.^4^ Yet, the phenomenological method seems at this point insufficient. What is needed is an investigation of the conditions under which the benefits provided by the society are produced. In the tradition of classical contract theorists, the argument is that constraints on the freedom of a person to act in self-interest can be justified by showing how the application of those constraints is beneficial for the person to whom constraints are applied. This goes beyond the phenomenological method, since it requires us to provide a justification of political obligations beyond the fact that people, as a matter of fact, comply with the obligations of their polities.

Moreover, given that even providing good reasons does not guarantee compliance, it is likely incentives in the form of penalties would need to be applied. The issue of compliance aside, however, Weale’s suggestion is that Horton’s phenomenology is a reflective method which is insufficient for the purpose of justification. Although reflective in character, Horton’s phenomenology is distinct from the method of reflective equilibrium, where an abstract theory derived through deductive reasoning is necessary; by contrast, Weale proposes an empirically based reflective equilibrium, which would find a place between Horton’s phenomenology and normative accounts, which are reliant on deductive reasoning. In the next section, some of the ontological presuppositions of Horton’s methodology will be examined.

## Ontology

The aim of Mendus’s paper is to examine Horton’s realist political theory, to investigate his critique of Rawls’s “high” or “liberal moralism” and to determine the extent to which, together with Horton, we would have reasons to leave Rawls’s and other Rawlsian accounts behind. The conclusion is that some of the insights of Horton’s realism are mistaken, whereas many of those which are not mistaken are compatible with liberal moralism correctly understood. Quite early on, the paper formulates the argument also in terms of contingency, in particular in terms of a contrast between the realist emphasis on the contingency of human existence and the liberal moralism’s neglect or inability to properly account for it, presumably due to a strong focus on necessity.

The paper begins with a brief presentation of the recent landscape in political theory, beginning with the bleak perspective it had in the 50s and 60s, its revival in the 70s, 80s and 90s starting from Rawls’s work, and continuing, mainly in the last 10–15 years, with the “realist” criticism of Rawls’s “ideal” theory. Next, Mendus presents the realist position, Horton’s version in particular. On some accounts (for instance, William Galston’s), realism sees Rawlsian liberalism as insufficiently political. One version of this realist criticism of liberalism is Horton’s emphasis on contingency, as an important aspect of political reality, which Rawlsian liberal moralism tends to overlook. Thus, on Horton’s account, liberal moralism is descriptively insufficient and normatively irrelevant.

In particular, Mendus notes, on Horton’s account, the acceptability of a political regime is a contingent and circumstantial matter. This appears as an important element of Horton’s theory of modus vivendi. As a result, Horton’s theory becomes both richer and more complex than liberal moralism. Moreover, his realism acquires a distinctive status: unlike the more aggressive and combative realisms of Machiavelli or Hobbes, which reject the priority of the moral over the political, Horton’s theory does draw inspiration from moral philosophy. In particular, Horton draws on the work of philosophers, such as Bernard Williams and Ludwig Wittgenstein, who do not reduce moral theory to the application of rules and principles, and argue for the importance of context in the discussion and evaluation of moral life.

The problem is, Mendus notes, that if Horton’s concern is with contingency, then it is not clear his main target should be Rawlsian liberalism. To show this, Mendus examines first the place of contingency in Rawls’s political liberalism. Secondly, she discusses the role of contingency in politics more generally. Finally, she offers a limited defence of moralism in politics. The first issue discussed is that of pluralism. According to Mendus, Horton implicitly criticizes Rawlsian liberalism for not taking pluralism seriously. On Horton’s account, the current pluralism of views on justice-related topics cannot easily be resolved. Moreover, in seeking to remedy one injustice, we may end up committing another one.

Mendus notes that Rawls insists both on the permanence of pluralism and on the inappropriateness of regretting the permanence of pluralism. In this context, one possible objection to Rawlsian liberalism is that it accepts permanent pluralism only for conceptions of the good, and not for those of right. Nevertheless, Mendus replies, even the acknowledgement of radical pluralism of conceptions of the good in society shows that Rawlsian liberalism takes pluralism very seriously. More generally, Rawlsian liberalism takes contingency very seriously. Thus, Mendus claims, Rawls’s Difference Principle starts precisely from an assumption of a contingent distribution of natural endowments, which indicates the difficulty of finding final answers to political problems.

Next, Mendus acknowledges that some political theories simplify too much and falsify the facts. Yet, she claims, the solution for this problem cannot be realism. One role for politics is to mitigate the worst effects of contingencies. On Rawls’s account, given the contingency of the distribution of natural talents, we can try to compensate for this contingency by giving moral meaning to these morally neutral facts. Thus, on the account offered by Rawlsian liberalism, the theory of justice is supposed to mitigate the worst effects of arbitrariness.

Another argument against the moralism of liberalism supports the claim that multiculturalist policies are continuous with the very racist and colonial policies they are meant to replace. This argument is similar in structure with the objection that liberal moralism ignores or is naïve about the realities of power in political life. Mendus is in agreement with Duncan Ivinson’s rebuttal: politics is not merely about power, but about the legitimate exercise of power. Hence, while it may be true that following principles in disregard of concrete contexts has damaging consequences, this does not show yet that realism is the solution. To insist in the realist manner that politics is mainly about power is to ignore the important issue of legitimacy and, hence, to be less realistic than realism claims to be.

The conclusion draws together the main arguments of the paper. First, Horton’s attack on liberal moralism is underpinned by a realism, which emphasizes the importance of context and circumstances, and the contingencies of the political life. This form of realism, however, can be distinguished from the classical forms, which are more combatively and aggressively opposed to the standard priority of morality over political theory; by contrast, the main aim of Horton’s realism is to convince the readers of the significance of his theory of modus vivendi – not through abstract reasoning, yet by reference to a moral theory which stresses the significance of the context and specific details, which are usually ignored by approaches which regard morality as the application of universal principles to particular situations.

Horton’s realism is a distinct type of political theory, but its emphasis on contingency is compatible with the strong emphasis Rawls himself places on the arbitrariness of the distribution of natural endowments. Moreover, Rawls’s view that one of the functions of political theory is to mitigate the worst effects of this arbitrariness takes into account an important feature of what politics is about. This is a feature Horton and other realist critics more generally would do well to take into consideration; to insist in the realist style that politics is about power would be to neglect one important part of the political reality, for which supporters of moral realism are so keen to account. In the next section, we move on to some possible answers to these and the previous objections formulated to Horton’s political theory.

## Modus Vivendi: Ontology, Method and Toleration

One of the aims of Horton’s paper is to encourage a different way of thinking about political theory. More exactly, the account which Horton puts forward is a distinct version of realism. Realism is a position critical of a form of political theory in which the ‘political’ becomes repressed or simply effaced. There are two related lines of criticism advanced by realism. First, there is a complaint that the conception of politics at work in liberal moralism lacks descriptive adequacy. Secondly, there is an objection that liberal moralism is utopian in a pejorative sense, and therefore largely irrelevant from a normative point of view.

The first complaint stems from liberal moralism’s lack of interest in the way political processes and institutions actually work, as well as in the obstacles that their effective functioning needs to overcome. For instance, political institutions are simply regarded as instruments constructed for the purpose of realizing antecedent moral principles or ideals. Moreover, political processes are discussed from the perspective of the assumption that individuals are free and equal, a claim which ignores the fact that people are always embedded in a variety of relations of power and only equal in a formal sense. For the realist, these power relations are not contingently related to politics, as it is shown by the focus of liberal moralism; liberal moralism concentrates on a too narrowly defined question, namely, the question of the end to which political power may be utilized.

The second objection stems from the idealizing assumptions of liberal moralism. For instance, Rawls’s assumption of strict compliance seems to go against the usual observations that even if Rawls’s principles of justice are right, there is little chance everyone would agree with them; even those who would agree with them might not interpret and endorse them in the same way; and even those who might endorse and might agree with their appropriate interpretation might not always act on them in particular circumstances. Politics is supposed to address these issues, rather than abstract from them with the assumption of perfect compliance.

Another issue, Horton notes, can be raised in relation to the objection that the problem of liberal moralism is not so much imagining the ideal it puts forward, but realizing this ideal. Yet, the problem goes in fact deeper – not only is it difficult to realize such an arrangement, but it is also difficult to imagine it. We would be more likely to imagine the view of a society where certain evils are avoided, but even in that case there might be disagreement about the things to include in this category. One further issue is the lack of an account of political agency in liberal moralist theories. Such an account could help with the examination of the issue of political change. Realists claim that, in making politics subservient to a philosophically constructed moral theory, liberal moralism misinterprets the relationship between morality and politics.

Horton tries not to dwell too much on the critical side of the realist position, and attempts to determine what realism proposes as an alternative to liberal moralism. Following Bernard Williams, Horton first claims that the political generates its own concerns and standards. The problem is the Hobbesian issue of order, but when Williams develops this insight, he obtains a principle which is close to the liberal principle of legitimacy. Following a more minimalist interpretation runs the risk of positing something too undemanding. The minimal moral universalism of John Gray or Stuart Hampshire suggests that there is a set of evils that need to be avoided, but this leads to the further problem of justifying these evils as evil. Moreover, sometimes it seems that political legitimacy can coexist with a failure to avoid these evils (for instance, torture). Finally, these evils and the goal of avoiding them seem to expand and become less minimal than initially presented.

Another key question concerns the cognitive and practical authority of the normativity of political theory over political agency. Without a justification of this authority, the views expressed by political theorists would represent only some additional voices in the debate. Furthermore, although in politics a topic often discussed is that of fairness, and although Rawls called his theory “justice as fairness”, no reference to Rawls’s theory is to be found in the pages of the tabloids or in the pages devoted to concrete policies. This suggests politicians and the general public are indifferent to political theory, in the way in which political theorists are indifferent to politics and politicians.

Some political theorists, Horton argues, who claim that it is important for political theory to be able to offer advice for concrete political issues conceive of this task as one of articulating ideals to be applied to concrete situations. Realists object to this approach, but when they offer something in turn there is usually disagreement between realists on the best approach to be pursued. The prescriptive realists agree with liberal moralists that political theory should be able to offer some guidance in concrete political situations, but they disagree with the way liberal moralists are trying to do this, and argue for a more practically relevant strategy, closer to the ethical and political complexities of political action in the ‘real’ world.

Horton is sympathetic with the approach of prescriptive realists, but does not endorse it; instead he endorses the approach of interpretative realists, who argue that political theory should be about understanding politics. This is not a merely descriptive enterprise, but although it involves an evaluative element, it does not include an aspiration to guide political action. Interpretative realism may have some effect on political action when this understanding is presented to the political actors, but whether, how and if it does this will be determined by political actors themselves, not by political theorists. Hence, realists should not seek to provide practical guidance. A political theory that aims at understanding and making sense of our political predicament, and the ways in which we think about it, including the role and meaning of political values, is a significant enterprise; it may also include reflection on the tensions and complexities of political life.

This approach is neither ideal nor prescriptive; it will be attentive to the circumstantial and contingent character of politics, it will try to understand the fundamental categories of political discourse and to understand the different ways of thinking about politics. This is not a purely descriptive enterprise: first, those who have tried to offer a purely descriptive account have failed, since even conceptual analysis cannot avoid at least some implicit, if only minimal, normative commitments; secondly, politics is bound up with the normative, and it becomes impossible for the theorist to entirely disengage from some degree of evaluation of the normative claims; even a theory devoted to understanding will be marked by normative inflections of various kind. There will be assumptions about how politics should be pursued, about better and worse scenarios, about political success and failure. This, Horton notes, does not undermine the distinction between a political theory aimed at understanding and one motivated by normativity.

In the final section of this paper, I will conclude by examining and evaluating the objections raised by Forst, Newey, Jones, Weale and Mendus from the perspective of Horton’s text.

## Conclusion

The discussion above interestingly shows one area of convergence for the objections to Horton’s account. This area of convergence is normativity. According to Forst, Horton’s account of toleration problematically asserts and denies that standards can have validity beyond reasonable disagreement. Thus, Horton claims that toleration is a moral virtue and, as a virtue, the objections to the practice or belief to be tolerated cannot be based on prejudice or hatred; instead, they must not be unreasonable or without value. At the same time, however, he thinks that disagreement on standards goes all the way down and affects all normative claims, and he does not take this to be a descriptive claim, but an implication of the limits of reason.

According to Newey, Horton’s account of toleration needs to include acceptance principles; the validity of these principles cannot be questioned counterfactually. At the same time, however, by advocating political arrangements in the form of a modus vivendi, Horton should also acknowledge that the actions, practices and beliefs of the members of a modus vivendi are not determined by principles, but by the play of the forces of those involved, as they are led by self-interests. By definition, then, a modus vivendi is not compatible with toleration, since in a modus vivendi agents do not have power to prevent what they object to.

For Jones, an important problem of Horton’s theory of modus vivendi is its normative status. On one interpretation, his modus vivendi is a better description of the political reality of modern democracies. On another interpretation, it is to be regarded as a model to be followed by liberal democracies. Moreover, a similar puzzle can be noticed in relation to the notion of legitimacy. On some interpretations, a legitimate regime is that accepted by the population. On some other interpretations, a legitimate regime is one which is deserving of compliance. If Jones is right and Horton has a normative notion in mind, then this goes against Horton’s scepticism about the normative role of political theory.

As Weale notes, Horton’s interest in the issue of political obligation introduces some normative constraints for which Horton’s political theory cannot account methodologically. What seems to be missing is an account of the reasons justifying to individuals why they should play their part in any scheme of cooperation necessary to produce the goods of the society (order and security). Because methodologically Horton’s account is to be understood as a reflective process on the content of ordinary moral consciousness, he cannot obtain normative reasons for participation. These, Weale suggests, could be provided by an additional method, which justifies constraints on a person’s freedom by showing how the application of these constraints is beneficial for the very person to whom constraints are to be applied.

Finally, Mendus focuses on Horton’s criticism of liberal moralism. One particular objection she considers is that liberal moralism ignores or is naïve about the realities of power in political life. Yet, she argues, one important element of political life is the legitimate exercise of power, where the notion of legitimacy has a strong normative content. Hence, to insist in the realist manner that politics is mainly about power and the contexts of power is to overlook the important issue of legitimacy. Therefore, in the attempt to be faithful to political reality, realism ends up further away from this reality, and turns out to be less realistic than it claims to be.

In the paper included in this special issue, Horton offers some resources for an answer to these objections. The crucial claim he makes is that his political theory is not meant to be non-normative, purely descriptive. It includes an evaluative element, but it does not set itself as a purpose the aim of providing practical guidance in politics. This indicates, first, that Horton’s theory is less vulnerable than it may have appeared at the beginning. He is not asserting a strong notion of normativity in order then to deny it, but includes from the outset an evaluative component in his theory. Moreover, he does not deny as a possible function for political theory that of providing guidance in specific political situations; he only is wary of theories that try programmatically to arrogate the task of providing advice.

Because he does not deny normativity, but only a particular picture of its significance, Horton’s account seems much stronger than initially presented. What may seem to remain problematic is the type of normativity acknowledged by Horton’s pragmatic account. On some of the construals presented above, Horton seems only open to a weak type of normativity, whereas his account demands a stronger one. The contrast usually drawn is between his view of normativity and that of Rawls, as the exponent of liberal moralism. This is indeed a contrast Horton himself acknowledges. But it is unclear how far Rawls’s own account of normativity is from Horton’s.

As noted by Jones, Rawls’s theory of justice is ultimately supported by ideas which are present in the public culture of democratic societies. Such a theory seems attentive both to context and detail, and brings Rawls’s account quite close to the realist camp. Remarks on the similarity between Rawls’s and Horton’s theories are offered also by Mendus, who notes the significance of contingency in Rawls’s view of justice, especially with regard to his view of natural endowments as arbitrarily distributed.

What is interesting, however, is that, despite these similarities, Forst, Newey, Jones, Weale and Mendus all claim (in more or less direct ways) that Horton should have offered a more robust account of normativity. Forst argues against Horton’s view of reasonable disagreement as applying to all standards. Newey notes the tension between a view of toleration which relies on a commitment to certain principles of objection and acceptance, on the one hand, and, on the other, an account of modus vivendi devoid of normativity. Jones is concerned with Horton’s scepticism about the normative role of political theory. Weale thinks Horton’s account must be supplemented methodologically in order to be able to provide justifying reasons for political obligations. Finally, Mendus thinks that the concern with a legitimate use of power cannot be addressed with the help of a realist account like Horton’s.

Yet, if we take Rawls’s theory as a paradigm of robust normativity, it is unclear whether Horton does not already have such a strong account of norms. After all, Rawls’s appeal to the ideas which are present in the public culture of democratic societies is quite similar to Horton’s reference to the content of common moral consciousness and to the constraints that one can formulate on this ground. To be sure, differences remain: Horton is suspicious of an attempt to ‘process’ those ideas from public culture in the way in which Rawls does (for instance, through the method of reflective equilibrium). But from a normative perspective this is only an indirectly relevant detail; the normative grounds of the evaluative claims of Rawls and Horton seem to be very similar. To be sure, even assuming this would be correct, there is a further question here whether this type of normative ground is sufficient, both within the framework of Horton’s account and more generally.^5^ This however will have to be the topic of another study.

## Footnotes

1 The papers in this special issue by Rainer Forst, Glen Newey, Peter Jones, Albert Weale and Susan Mendus are clear illustrations of the significance of his contributions to these current debates.

2 Hereafter, PC.

3 Towards the end of the paper, Newey formulates the PC to take into consideration all these objections, as follows: **A** can only tolerate a disapproved-of action or state of affairs **P** if *either* (i) **A** believes that it is within his power to prevent **P** and (ii) it is not the case that **A** would seek to prevent **P**, if **A** believed he had the power to do so, even if, in **A**’s judgement, prevention did not incur undue costs; *or* regardless of (i) and (ii), **A** accepts reasons for non-prevention over and above those of practical feasibility and cost.

4 As Weale correctly notes, however, having such a reason does not mean that the individual member of society will comply. The question of compliance is a different question from that of justification.

5 This is a question Forst, too, raises in his contribution to this special issue.

